# Audit of donor centre: guidelines by the World Marrow Donor Association Quality and Regulation Working Group

**DOI:** 10.1038/s41409-022-01563-3

**Published:** 2022-01-14

**Authors:** Maren Weber, Nicoletta Sacchi, Sherry Haun, Ingrid Tistl, Stephanie Thompson, Hirasine Sengomona, Salmah Mahmood Ahmed, Oliver Kürsteiner, Carolin Schwarz, Jennifer Wuchter

**Affiliations:** 1 DKMS gemeinnützige GmbH, Tuebingen, Germany; 2 Italian Bone Marrow Donor Registry-Registro nazionale Donatori di Midollo Osseo, Genova, Italy; 3 Canadian Blood Services, Ottawa, ON Canada; 4 ZKRD Zentrales Knochenmarkspender-Register für die Bundesrepublik Deutschland gemeinnützige GmbH, Ulm, Germany; 5 NMDP/Be The Match, Minneapolis, MN USA; 6 Anthony Nolan, London, UK; 7 Blutspende SRK Schweiz AG, Bern, Switzerland; 8 DKMS Registry gGmbH, Tuebingen, Germany

**Keywords:** Haematological diseases, Health care

## Abstract

According to the Standards of the World Marrow Donor Association (WMDA) 2020 [1] unrelated stem cell donor registries are responsible for compliance of their donor centres with these Standards. To ensure high stem cell product quality and high standards for safety and satisfaction of voluntary unrelated stem cell donors, we present here guidelines for audits of donor centres (DC) that can be used by new and established donor registries. They have been developed for registries relying on independent national or international DCs for the recruitment and management of Unrelated Donors (UD) for verification typing (VT)/extended tying (ET), work up processes and Hemopoietic Progenitor Cell (HPC) donation. The main goal of these guidelines is to support registries in verifying and auditing their affiliated DCs to ensure they are compliant with the WMDA Standards, as well as WMDA recommendations. We define the general requirements and recommendations for collaboration with the DC and guidelines to manage the UD, step by step from recruitment to follow-up. We also provide a checklist, intended to serve as a resource for auditors performing an audit at a DC.

## Introduction

This document was prepared by the World Marrow Donor Association (WMDA) Quality and Regulatory Working Group (QRWG) and approved by the WMDA board. It contains the guidelines for audits of (DCs) by unrelated Donor Registries (DR).

WMDA ed. 2020 Standard 1.06 states: [https://wmda.info/professionals/quality-and-accreditation/wmda-standards/] “If a registry relies on an independent donor centre or cord blood bank to recruit and characterise donors/umbilical cord blood units, the registry must ensure that the donor centre/cord blood bank complies with relevant WMDA Standards. The nature of these affiliations and the duties and responsibilities must be documented in an agreement.”

Registries are responsible, indirectly, or directly, for ensuring the right donors are recruited, managed and protected at all times before, during and after HPC donation, based on agreements with DCs.

These guidelines have been developed for registries relying on independent DCs, located in the same or in a different country.

The main goal of these guidelines is to support registries in verifying and auditing their affiliated DCs to ensure they are compliant with the WMDA Standards, based on the 2020 edition. This means that the authors interpreted the current standards and deduced specific process steps and requirements for DCs.

Registries should be aware that these guidelines may not cover all aspects necessary to comply with local governmental laws and regulations. It is therefore the responsibility of each registry to determine and follow any additional governmental regulations and guidelines applicable to them, and they are also responsible for adapting the checklists to meet these specific requirements.

In some countries it may be the responsibility of the DC to ensure compliance with local governmental laws and regulations.

These guidelines and related checklists lay the groundwork that should apply in most countries worldwide and help registries to ensure the WMDA Standards are adhered to by their DCs.

These guidelines do not supersede established quality and regulatory systems, but instead endeavour to embed these in the framework of donor management and ensure that the current WMDA Standards, as well as WMDA recommendations, will be followed by the DCs. If the DCs have any other accreditation, such as FACT (Foundation for the Accreditation of Cellular Therapy)—JACIE (Joint Accreditation Committee ISCT-EBMT), AABB (formerly known as the American Association of Blood Banks), (EFI) European Federation of Immunogenetics or (ASHI) American Society for Histocompatibility and Immunogenetics this should be demonstrated in their quality management system.

As standards and requirements change in the future, registries are advised to make proposals for changes and additions.

### Format and timing of audits

From UD recruitment, consenting, testing, management, and the collection of donors personal, genetic, and medical data, the essential procedures rely on high-quality standards at DCs. As the DR provides the volunteer UD with all necessary information about their role as an UD, the DR is also responsible for ensuring that they comply with WMDA Standards as a minimum requirement.

Regular audits provide the means of monitoring compliance. The DC should embed these audits within their own quality system. The following formats and frequencies of audits are recommended.

It is recommended that a full audit is conducted, including an on-site or remote/virtual inspection of any DC that is to be newly contracted by a registry. During the application period, all national and WMDA requirements must be considered. If it is not possible to conduct the audit on-site, a remote audit can be considered, provided that the technical and data protection criteria are met. Additionally, a paper-based audit is required to ensure compliance with the WMDA Standards.

For already contracted, experienced DCs, a biennial paper-based audit should be conducted by the DC, reviewing at least the following points:

- Change of address or other important contact information to ensure that all critical communications with collection centre (CC), transplant centre (TC), and UD can be accomplished in a timely manner.
- Relevant staff changes (e.g., Medical Director, CEO, physicians, or coordinators).
- Changes in accreditation, registration, or license status.
- UD issues which may include the number of serious adverse events and serious adverse reactions occurring and reported to the DR.
- Changes in suppliers, testing laboratories or other affiliated facilities, if required by the DR.

It is recommended that once every 4 years the DR performs a full on-site audit based on these guidelines and the provided checklists. Whenever a DC fails to meet the basic requirements, immediate clarification must be made and both corrective and preventative actions must be taken by the DC.

The DR should have a mechanism to re-audit the DC in 6 months—1 year to ensure that the corrective action plan/preventive plan was functioning as intended.

If the DC fails to comply with the request in the time allotted, the DC activity can be either suspended, or withdrawn.

Each registry should establish its own auditing group of staff fully trained in audit procedures.

### General organisation of a DC

It is important to have a clear and defined organisational structure as this provides the basis for a quality programme and ensures the structure reflects the needs of the registry, its affiliations, and customers. The general information includes the DR’s organisational structure and the basis of its operation. Cooperating affiliates such as DCs, testing laboratories, (CC) etc. are functionally part of the DR’s performance and are required to comply with WMDA Standards.

There needs to be a sufficient number of trained personnel to carry out DC operations, and assurance that staff are qualified for the tasks they perform. Qualifications are identified based on job responsibility as well as assigned tasks. Staff members must be made aware of the sensitive nature of their job, and the importance of confidentiality must be defined in policies and procedures, as per national regulatory and applicable standard requirements. It is important to ensure that staff are trained to maintain anonymity of the potential donors and transplant—patients and rules of disclosure must be established.

There are many medical issues that can arise during the day-to-day activities within a DC, including donor health/eligibility issues, unusual (TC) requests or complications during the stem cell collection process. There should be at least one designated physician to review these issues to provide guidance and direction regarding each case. These physicians should be experts in the field of stem cell transplantation and histocompatibility and should have a broad familiarity with general medicine, in accordance with national regulations and registry specific requirements.

Management of key DC processes is achieved through process control methods such as standard operating procedures (SOPs). Specific performance goals should also be identified and reviewed, and the DC should be able to demonstrate that enough people are working in each area to meet these goals. Mechanisms should also be in place to monitor key performance indicators (KPIs), as this is important for identifying any changes that may be required in staffing or processes.

Selecting dependable, quality suppliers who provide services to support DC functions is essential. A system of review and monitoring is important to assess and ensure conformance to the standards, regulations, policies, and procedures. Monitoring and review methods are determined between the DR and DC and should include practices such as internal/external audits, laboratory proficiency testing, outcomes data collection, review of quality indicators and feedback from customers and other stakeholders.

### Cross border DCs

Some DRs may also support DCs located in a different country. These collaborations may range from providing infrastructure and support for international transmission of requests and testing results to supporting the international centre in various aspects of donor management or coordination procedures. DC auditing is not intended for situations where one DR strictly hosts the donor database of a different DR (guidance to WMDA Standard 1.01). These collaborations should be evaluated on a case-by-case basis and compared to the relationship with national DCs, since in the case of international affiliated DCs, challenges can arise due to different national regulations, communication (different languages, different cultures), unclear lines of authority and variation in how responsibilities are shared if there are several affiliated entities. International partnerships may also necessitate additional auditing of, for example, national donor recruitment requirements and consent, standard infectious disease markers (IDMs) and medical testing requirements, routine medical examinations or data security and confidentiality if these differ in the other country. Therefore, these audits and checklists may vary from those of national DCs depending on the conditions of the individual cooperation and similarities or differences between operations and regulations in the countries involved.

### Key performance indicators

The KPIs represent a quantitative assessment of key activities of a registry, which in turn reflect the activity of associated DCs. Their main objective is to improve processes by defining achievable target values which results in safe and efficient services for transplant recipients. These KPIs are based on the WMDA annual report and the Human Leukocyte Antigen (HLA) discrepancy report as evaluated each year by the Working Group Quality and Regulation.

By setting KPIs for participating institutions (registries and DCs), the WMDA aims to improve the quality of processes. There should be procedures in place for establishing and evaluating the institution’s KPIs in relation to the WMDA recommendations, as well as for analysing associated procedures if shortfalls are identified, to determine a suitable action plan. The institution’s KPIs can also be compared to the data of other institutions of similar size (see WMDA report). Results and evaluations should be documented. Monitoring of KPIs should be on an annual basis since results are collected and published by the WMDA annually. Further information can be found on the WMDA website [https://wmda.info/professionals/optimising-search-match-connect/tools-to-operate-a-registry/].

### The audit of management of the donor step by step

#### Recruitment

There is some variability of donor recruitment process between registries, including the target groups they may focus on. Some countries have government regulations that guide recruitment activities while others have policies and procedures that indicate how donors are recruited. According to WMDA Standards, the recruitment of donors must be performed by professionals trained for recruitment, under the direction of individuals who are experienced in recruitment of donors and in management activities including education, consenting, counselling, confidentiality, and medical screening [1].

#### Procedure for donor management at time of registration. SOPs, policies, and recruitment material

A procedure which clearly outlines the responsibility and tasks of the DC for donor recruitment activities must be in place. Effective management of documents, including SOPs, manuals, recruitment materials, and records, ensures staff are performing activities according to defined requirements and demonstrates ability to record, trace and retain information in various forms.

It is important to confirm how essential documents are controlled and managed to ensure staff have access and are trained to the most current versions. This is especially important for critical processes such as management of donor consents and samples to ensure privacy, confidentiality, integrity, and traceability. For example, labelling of donor samples is vital to ensure the correct linkage of the donor’s personal data with the corresponding sample sent for HLA-typing.

For these reasons the DC audit must assess if the DC has a defined process for recruitment of donors, and the management of consents and samples at the time of recruitment.

#### Consent forms

When recruiting donors, it is important to ensure they have received appropriate counselling and provide the necessary informed consent. Informed consent is to be written according to national laws and regulations and must adhere to WMDA Standards as well as registry policy [2]. Donors must sign/electronically submit a consent form at the recruitment stage. Written information must be given out to reinforce other forms of communication.

There should be a written policy in place outlining the elements to be included as part of the donor counselling process. The policy should include the importance of donor commitment and the obligation involved when registering as a donor, including the time required to complete the donation process and potential financial impacts if the donor does not receive payment from their employer for time they are absent from work. The WMDA provides specific guidance to be included [https://wmda.info/professionals/quality-and-accreditation/wmda-standards/], [2, 3], as part of the counselling and consent process and this should be used as a reference tool while reviewing completed consent forms. Recruitment brochures and other forms of written material provided to the donor at the time of recruitment should also be reviewed, including confirmation that the most current version of recruitment material is used.

The focus of the audit is, therefore, to assess that the DC conveys required information at the time of recruitment to ensure informed and committed consent.

This can be done by review of selected consent forms completed by donors recruited in the last 4 years.

#### Assess rules for donor eligibility, age, and gender

A policy should be available which outlines information to be collected from a donor at the time of registration and this must include donor age and gender as per WMDA requirements. Tools such as a medical health questionnaire or checklist may also be used to capture this information. DC staff should have access to guidance material which defines and clarifies all medical criteria that would exclude an individual from joining the registry based upon safety considerations for the donor, as well as considering safety of the patient. The WMDA provides minimum standards for which potential donors should be assessed [4].

Additionally, there should be a policy in place describing the circumstances for when a donor may receive the results of their health screening and the method of communicating these results to the donor must be appropriate to the situation based on privacy, regulatory and/or legal requirements where applicable.

#### Assess the DC has an established procedure for the management and storage of donor data

Each country has its own laws and regulations relating to privacy, confidentiality, and the protection of personal data. Policies and procedures regarding information management should be in place which indicate how data are captured/entered by DC staff and how the data are verified, saved, and transferred to the registry database.

It should also be confirmed that access to electronic data is limited to authorised individuals. Since the search for an unrelated donor is an anonymous process, policies and procedures must be in place to ensure donor confidentiality, including the assignment of an anonymous donor identifier.

#### Assess training for donor recruiters

Recruiters are often the first point of contact for potential new registrants and have an important role in educating potential registrants as well as ensuring availability and commitment of registered donors. Due to the important work involved with stem cell recruitment, a rigorous training programme should be in place for recruiters and recruitment supervisors. The WMDA has developed recommendations [1] for minimum qualifications and training of stem cell donor recruiters that should be referenced as a guideline for management of recruitment personnel. There must be a means of clearly capturing training requirements for each role as this ensures that those performing the specific requirements for that role consistently meet training requirements e.g. training matrix. Documentation should also be available for training performed at the time of hire and on an on-going basis.

### Verification typing/extended typing (VT/ET)

Auditing of the VT process should include all aspects of the process: from maintenance of appropriate policies on suitable donor counselling, maintaining confidentiality, adherence to the respective health screening and testing requirements, correct sample collection, labelling and shipment procedures, as well as timely and accurate processing of requests and transmission of results. Donor screening and testing, as well as questionnaires designed to detect and evaluate conditions that increase risk of donation, can be found in WMDA guidelines and the donor medical suitability website [https://share.wmda.info/display/DMSR/WMDA+Donor+Medical+Suitability+Recommendations+Main+page], U.S. Food and Drug Administration regulations, AABB (formerly known as American Association of Blood Banks) and FACT-JACIE International Standards.

#### Procedure for donor management at time of ET or blood sample collection for VT

If a potential stem cell donor is found to be a good match for a patient, the DC must provide the donor with appropriate information and material(s) and take steps to counsel the donor regarding the need for additional testing. To achieve the donor’s full comprehension, this information must be provided in the national language. The DC must ensure the donor is making an informed decision, based on what is being asked of them.

At a minimum, the DC is expected to have a written policy that documents the following elements when counselling the donor:

- Anonymity of the donor and patient, the confidentiality of personal data
- Donation is for any patient in need, including an international patient
- Donation not being remunerated
- Requirement for further blood samples before donation
- Requirement for infectious disease and other testing (e.g. HLA), which testing will be performed, that results will be communicated to the TC, and the possibility that samples may be retained for further testing
- Implications of transmission of infectious and other diseases from donor to patient
- Principles and risks of donation
- Possible duration of loss of time from normal activities
- Location of the collection
- Potential for collection of autologous blood
- Right to withdraw and consequences for the patient
- Details of insurance coverage
- Possible subsequent donations of hematopoietic stem cells (HSC) or blood products
- Alternative collection methods and whether blood is reserved for research purposes.

Information must be appropriately documented and retained according to local requirements.

#### Request for blood sample collection for VT/ ET

The DC must have a well-defined policy that preserves donor confidentiality, as the identity of the donor must be protected from disclosure. Centres should avoid the use of donor and patient names in communications and solely use a donor unique identifier preferably the Global Registration Identifier for Donors (GRID) wherever possible [5].

The DC shall have a policy that provides direction for managing confidentiality compliance with all records and communications relating to donor and patient. The communication between parties involved with donor information must be traceable on all pages of the records and source documents. Personnel performing respective operational tasks must be identifiable and have documented training.

#### Medical questionnaire completed at time of VT

The medical questionnaire is used as part of the process to evaluate a donor’s suitability and eligibility status for donation. At the time of VT, the medical history questionnaire should provide information that evaluates a prospective donor’s suitability and eligibility status for donating stem cells. This includes donors that convert from peripheral donation to bone marrow harvest and vice-versa. Each questionnaire should target a specific reason for excluding or deferring the donor from donating. They should gather information about risk behaviours that may have exposed the donor to certain diseases. This is a critical aspect because discovery of new risk findings necessitates that the potential donor might require further evaluation or deferral. For example, since the bone marrow donation is a surgical procedure that requires an operating room, additional questions may be required for a donor response that indicates possible complications with anaesthesia from a previous procedure. Also, such information or other possible identified risks could impact product selection (marrow collection vs. apheresis) or the health of the recipient, which the TC would need to be informed about at the VT stage.

The interview is one way to assess a donor’s risk for an infectious disease, however, it is mainly dependent on the interviewer’s appropriate knowledge and use of tools, and the donor’s comprehension of the questions. It is essential that the DC accurately determines the donor health status to protect them from risk of damage to his/her own health for the donation and to protect the patient from transmission of communicable diseases. It is especially important to identify potential risk for diseases and conditions for which there are no adequate laboratory tests or for which tests are unable to identify early stage or window period infection. Assessing the risk of disease transmission may identify behaviours associated with risk of disease transmission. Positive responses on a screening questionnaire may lead to more in-depth donor evaluation and a thorough assessment of risk versus benefits, or donor deferral.

#### Accompanying documents for shipment of blood samples

The blood sample must be labelled according to national specifications, and at a minimum should include a donor identification number/GRID and information on the type of sample (e.g. anticoagulants/additives) as well as the collection date. However, the amount of information which can be included on a label is limited by space. Additional or more specific information, as well as patient and TC identification, may therefore be included in accompanying documents sent with the sample. This may also include additional forms used for traceability or documentation that the centre collecting the blood sample uses as distribution records.

#### Process for updating donor status and HLA at VT

For the patient to have the highest potential of a successful transplant, the donor’s HLA must be closely matched with the patient. Preliminary searches may identify just a few or many potential matches. HLA matching depends on the level of DNA-based resolution typing (low, intermediate, high) and on which loci are tested. Once a donor is selected as a potential request for a specific patient, the donor should become temporarily unavailable for other potential patient and should not be represented on TC search reports, or alternatively be marked as reserved.

Additionally, upon receipt of the VT result, this needs to be compared to the previously listed donor HLA. Procedures should be in place for updating donor HLA according to the respective policy (e.g. how the DC deals with results at more loci and higher resolution). Procedures also need to be in place for investigating any HLA discrepancies which may be detected after receiving results from the TC.

#### Infectious disease markers and blood group results

The purpose of IDM testing is to assess the donor’s exposure to infectious diseases and the likelihood of transmitting a disease to the patient. It indicates if an individual currently has, or previously had, an infectious disease that could be transferred to another person. Routine health screening and personal history and physical examinations are not sufficiently comprehensive evaluations for hematopoietic cell donations. There are numerous infectious agents that, if present in the donor, pose definite risk to the transplant patient and are only detected via IDM testing. Testing must be performed to meet the minimum requirements of the WMDA [https://wmda.info/professionals/quality-and-accreditation/wmda-standards/]. The geographical location or other local factors may require additional testing according to national requirements as well. WMDA Donor Medical Suitability [https://share.wmda.info/display/DMSR/WMDA+Donor+Medical+Suitability+Recommendations+Main+page] provides additional information on testing at VT.

Policies need to be in place specifying which testing is to be done at VT and which information is transmitted by which means to the TC and that all required testing according to WMDA Standards and national requirements at a minimum are performed and communicated accordingly. Procedures must be in place to inform donors and TCs accordingly if positive IDM results are detected which could have implications on donor or recipient health.

### Work up

#### General requirements

Donors are healthy individuals who are acting altruistically and willing to take some small risk to help others. Donors are volunteers and can withdraw from the process at any time; no undue pressure should ever be applied. The process toward the donation is very special for the donor as well as for their family and friends. Therefore, it is recommended that the staff at the DC and CC is prepared to handle the donor with appropriate sensitivity and to make them feel special and well cared for during the entire workup and donation process.

A policy that clearly establishes the responsibility and tasks of the DC and CC must be in place. The SOP must ensure proper planning and coordination of appointments and communication with the TC during the entire workup procedure.

WMDA Standards and guidance are well detailed in describing the requirements for this very critical and important process and serve as an important reference for setting up an audit focused on recognition, documentation, and appropriate action in relation to the workup process.

#### Workup process: from information session to product collection

The donor management at time of workup must be performed according to National laws, regulations, and WMDA Standards.

The DC should have a SOP in place to define the process of organising the work-up, in collaboration with the CC. These procedures must include the process to verify the prescription, inform and counsel the UD, and to test the UD including medical history, PE, and laboratory tests to determine the UD’s fitness to donate. Workup request forms of the registry are expected to include information such as date of patient’s conditioning and graft infusion, proposed collection dates, and donor clearance date.

The purpose of VT is to ensure that the individual being selected for donation is the same individual whose HLA typing was listed on the search report used to select the donor. A process must be in place to ensure the DC receives and verifies the results of VT/ET before the activation of a workup. The VT must be performed at a minimum of HLA-A, -B, -C, -DRB1 and must comply with registry SOPs before processing the workup request.

There must be policy/procedure in place to confirm that prescription forms are detailed, comprehensive, include clear donor identification (donor unique identifier/GRID), patient status and degree of urgency.

According to WMDA Standards and guidance there must be a policy to inform the TC/registry of critical information pertaining to the donor which could impact the collection or transplant. For example, if the donor cannot be reached in a reasonable number of days after the workup request, or when complications occur. There must also be a written policy that describes how the TC is advised of donor preferences and other related issues, such as the release of products that are considered to have increased risk. Refer to WMDA guidance for further examples.

There must be a process in place to verify with the CC the workup request and prescription, including the timing to send back to the TC the signed prescription form (verification of prescription/cell product).

#### Pre-donation information session/donor informed consent

A written policy must be in place to obtain a valid signed informed consent from the donor. According to WMDA Standards the informed consent form must be written according to national laws and regulations and must adhere to WMDA Standards and registry SOPs.

The responsible DC physician or their designee, in accordance with the responsible CC physician, must explain the procedure in a language and terms the donor can clearly understand, and at a minimum the information session must cover the topics as listed in WMDA Standards and guidance.

A procedure must be in place to verify, with the CC, the identity of the volunteer donor, at a minimum at workup and at collection, by the qualified staff signing the consent form. The responsible DC physician or their designee must be aware that the UD can decline the donation process at any time; and training should be provided for managing this situation, including the requirement to inform the TC/requesting registry immediately if the UD has declined to proceed with the donation.

A policy must be in place to retain consent documents signed by volunteer donors in a secure way to protect confidentiality. Consent forms must be promptly available for review by individuals designated by the registry or national authorities to evaluate the registry.

#### Collecting pre-collection samples

As per the WMDA Standards, DCs must have the capability of shipping samples, if requested, to the facility indicated by the TC if required for further testing. The sample must be appropriate for the testing required. A policy should be available which outlines how and when to collect a pre-collection sample. In particular, the donor must sign the informed consent form before any pre-collection samples can be collected, and the DC must ensure the identity of the donor before collecting the samples e.g. photo ID.

The samples must be prepared for shipping according to international safety recommendations, national rules, and laws and according to the shipping instructions provided on the workup forms.

The samples must be labelled according to national regulations, WMDA Standards and accreditation of the facility. National requirements as well as the registry’s policies concerning the maximum volume of peripheral blood to be collected from an UD at the time of physical exam must be followed. In a case where there is more blood requested from the UD than usual, the responsible DC or CC physician or their designee can decide to decline additional blood collection for donor safety reasons. This decision should be reported to the TC/requesting registry immediately.

#### Medical assessment and unrelated donor eligibility

If a registry/DC relies on an independent CC for the collection of donor haematopoietic stem cells or other donor samples, for donor medical evaluation or for the follow-up of donors, the registry/DC must ensure that the CC complies with WMDA Standards in these areas. Detailed requirements are reported in the WMDA guidance [6].

The DC together with the CC are responsible for planning the medical assessment of the UD which must be performed according to national standards. WMDA Standards should also be applied at a minimum. The DC and CC must ensure the medical and physical examination of the donor is completed and recorded. This examination must be performed or supervised by a physician who is not a member of a team who has cared for the patient. The DC must have a plan to select a different physician or CC in order to comply with this requirement

The communication policy/procedure must include steps required, including immediate communication to the TC, if any issues arise that could impact the collection schedule

SOPs should be in place to cover the following:

- Possible need for central venous access
- Mobilisation therapy for collection of HSC and apheresis
- Anaesthesia and HSC bone marrow collection
- Pregnancy assessment for all female UDs according to national standards and regulations.
- Infectious disease markers (IDMs) must be tested within 30 days prior to the HSC collection or according to the applicable law and national requirement as well as to WMDA Standards. If country-specific IDMs are required (by applicable law and/or regulations), the UD must be tested for evidence of clinically relevant infections accordingly.

IDM testing must be carried out in a manner to ensure the safety and accuracy of the data by laboratories that meet national requirements and standards (as well as international lab standards if applicable), and using diagnostic tests approved by the government or prevailing authority in the relevant community for performing these services. Additional tests must be performed as required to assess the possibility of transmission of other infectious or non-infectious diseases. UDs who have recently travelled outside their country should be evaluated for infectious agents prevalent in the areas of travel. The DC must have a procedure to counsel the UD in case of positive test results.

#### Unrelated donor final clearance

After having collected all required information about the UD’s health, the responsible DC and CC physicians or their designee determine if the UD is suitable for the requested collection procedure and this decision must be documented on the final clearance form according to the WMDA Standards and guidance. The DC and/or CC physician or their designee is responsible for completing and signing the clearance form prior to collection. Even if not required by national laws or requirements, subsequent review by a second person is strongly recommended. The donor final clearance must be communicated in writing to the TC before commencement of patient conditioning and before the UD begins the mobilisation regimen. Internal policy must also ensure that the DC/CC clearly communicates the expected hour for end of collection, the pickup address for the product as well as CC contact information.

The donor final clearance and the detailed results of the medical assessment as well as the signed informed consent form from the UD must be added and stored with the donor records for at least 30 years.

#### Medical ineligibility

A policy must be in place which clearly states what to do in case of medical ineligibility of the UD for the requested collection procedure. The registry standards and national regulations must be followed; additionally, WMDA donor suitability as well as registry-specific medical eligibility criteria can be consulted for general recommendations [https://share.wmda.info/display/DMSR/WMDA+Donor+Medical+Suitability+Recommendations+Main+page]. The DC/CC must have a procedure in place to inform the TC immediately regarding medical ineligibility of the UD. Additionally, this information must be forwarded to the registry in writing using WMDA forms or equivalent.

When a potential risk for the patient is identified, there may be a need to declare the UD as ineligible for the patient according to national requirements and laws. The TC is to be informed via the responsible registry according to the applicable data protection laws and requirements using the WMDA form (or equivalent).

Additionally, some country-specific requirements must be fulfilled e.g. declaration of medical need, declaration of ineligibility. These documents must be included in the donor records and provided to the responsible registry. UD eligibility can only be confirmed once the TC has evaluated the risk and deemed acceptable to proceed. Additional information about the health of the UD that might be relevant for the TC but does not have a direct impact on the UD’s eligibility, can be included as comments on the WMDA form or equivalent.

#### Insurance coverage for the donor

It’s important to ensure information is provided to the donor about expenses and disability and death benefits for volunteer donors. The DC should have a policy in place to inform the donor regarding extra expenses and insurance coverage

### Follow up

The registry must have a process in place to provide donors a method to report post donation related medical concerns. Post donation, it’s recommended the registry follows the donor a minimum of 10 years to monitor for potential medical issues.

### Serious adverse reaction/serious adverse event (SAR/SAE)

Donors are healthy persons who voluntarily donate for a patient and are accepting some small risks in order to help others. Therefore, although the risks for both parties are clarified before donation/transplantation and are generally minor, it is still important to monitor the health and well-being of the donor not only during the collection procedure, but also afterwards.

The intent of WMDA Standards is to ensure that measures are in place to assist in detection and reporting of the occurrence of a SAE and a SAR, and that should a SAE or SAR occur, that the donor can be monitored and treated appropriately, and that all parties involved are suitably informed so that the TC can also care for the recipient appropriately, if necessary.

Additionally, the WMDA reporting system (S(P)EAR) can assimilate long-term data on possible risks associated with the donation process, as well as assist in the (rapid) distribution of SAE-/SAR-related information to registries and their partners to assist in the prevention of the recurrence of such events.

The focus of the audit is, therefore, to ensure procedures exist for the recognition, documentation, and appropriate action plans in relation to SAEs and SARs, examples of documentation and investigation of SAR/SAE, and initiation of necessary remedial and/or corrective action

Both SAEs affecting a cellular product intended for a specific patient as well as SARs affecting donors undergoing collection of HPC and/or cellular product must be identified, documented, investigated and remedial and/or corrective action taken.

Procedures should also include the system of documentation of cases and corrective action and may also describe who processes and reviews cases. DC must have a procedure in place, which describes how and when cases are reported to the registry or directly to the WMDA.

### The checklists

The checklist in the appendix is intended to serve as a resource for auditors performing an audit at a DC. It is intended to enable the auditor to perform an audit using the checklist only. The checklist mentions the relevant WMDA Standards and refers to the chapter that should be covered by the audit. The auditor is also provided with clear information on which files should be inspected and which processes should be verified. Depending of the type of the UD’s affiliation (only working with local DCs or cross border DCs or working with a combination of both); the checklist may be used on its own, combined with the addendum checklist with specific points for cross border DCs.

## Conclusion

The DR is ultimately responsible for ensuring its affiliated DCs, whether local or international are compliant with the WMDA Standards; this is to ensure the safety of unrelated adult donors and to make their donation journey the best experience possible.

It is strongly recommended that DRs and their affiliated DCs have well established policies and procedures, using the recommendations of the WMDA and outlining the roles and responsibilities of each party, to support unrelated adult donors from the recruitment process, donor selection and collection to donor follow up as well as incident reporting. DRs need to ensure DCs are well equipped to carry out these activities.

When collaborating with cross border DCs, DRs should be aware of laws and regulations governing the country of each DC.

## Supplementary information

Supplementary Data 1

Supplementary Data 2
